# Oral delivery of anti-TNF antibody shielded by natural polyphenol-mediated supramolecular assembly for inflammatory bowel disease therapy

**DOI:** 10.7150/thno.47601

**Published:** 2020-08-29

**Authors:** Xinyu Wang, Junjie Yan, Lizhen Wang, Donghui Pan, Yuping Xu, Fang Wang, Jie Sheng, Xinxin Li, Min Yang

**Affiliations:** 1 NHC Key Laboratory of Nuclear Medicine, Jiangsu Key Laboratory of Molecular Nuclear Medicine, Jiangsu Institute of Nuclear Medicine. Wuxi 214063, China.; 2 Department of Radiopharmaceuticals, School of Pharmacy, Nanjing Medical University, Nanjing, Jiangsu, 210029, China.

**Keywords:** Supramolecular nanoparticles, oral delivery, polyphenol, anti-TNF therapy, inflammatory bowel disease

## Abstract

**Rationale:** Anti-tumor necrosis factor (TNF) therapy is a very effective way to treat inflammatory bowel disease. However, systemic exposure to anti-TNF-α antibodies through current clinical systemic administration can cause serious adverse effects in many patients. Here, we report a facile prepared self-assembled supramolecular nanoparticle based on natural polyphenol tannic acid and poly(ethylene glycol) containing polymer for oral antibody delivery.

**Method:** This supramolecular nanoparticle was fabricated within minutes in aqueous solution and easily scaled up to gram level due to their pH-dependent reversible assembly. DSS-induced colitis model was prepared to evaluate the ability of inflammatory colon targeting ability and therapeutic efficacy of this antibody-loaded nanoparticles.

**Results:** This polyphenol-based nanoparticle can be aqueous assembly without organic solvent and thus scaled up easily. The oral administration of antibody loaded nanoparticle achieved high accumulation in the inflamed colon and low systemic exposure. The novel formulation of anti-TNF-α antibodies administrated orally achieved high efficacy in the treatment of colitis mice compared with free antibodies administered orally. The average weight, colon length, and inflammatory factors in colon and serum of colitis mice after the treatment of novel formulation of anti-TNF-α antibodies even reached the similar level to healthy controls.

**Conclusion:** This polyphenol-based supramolecular nanoparticle is a promising platform for oral delivery of antibodies for the treatment of inflammatory bowel diseases, which may have promising clinical translation prospects.

## Introduction

Antibodies have emerged as one of the most promising classes of drugs due to the tremendous success in the treatment of various diseases, including cancer 1, autoimmune 2, cardiovascular 3, infection 4 and so on. Infliximab (INF), adalimumab, golimumab, and certolizumab pegol are antibody therapeutics for the treatment of inflammatory bowel disease (IBD), which is an incurable chronic disease 5. These antibodies inhibit tumor necrosis factor (TNF) alpha, the main pro-inflammatory cytokine secreted primarily by macrophages during IBD 6. The robust efficacy achieved in patients by anti-TNF agents has changed the way of treating IBD refractory to conventional medications, such as corticosteroids and immunomodulatory. Despite the many advantages of anti-TNF therapy, there are still many deficiencies. Nearly half of the patients do not respond to the anti-TNF therapy 7. Furthermore, the patients received anti-TNF therapy may suffer the serious adverse effect, such as the increased risk of tuberculosis 8, malignancies, and serious infections 9, because of the systemic immunosuppression by systemic exposure to antibody. Due to immunogenicity of the drug, response failure is not uncommon in responding patients 10. Anti-drug antibodies were found in 10-20% of patients receiving anti-TNF maintenance therapy, resulting in response failure 11.

The ideal anti-TNF therapy for IBD should deliver the antibody directly to the sites of intestinal inflammation so that systemic exposure and immunosuppression can be avoided. Currently, antibody drugs are administrated parenterally, whether subcutaneously, intramuscularly, or intravenously 12. Oral delivery is the most common method of drug administration with high levels of patient acceptance and the potential to deliver antibody for gastrointestinal (GI) diseases. It is reported that IgA from maternal milk is a critical factor in preventing the development of necrotizing enterocolitis in preterm infants 13. AVX-470, an orally delivered antibody with anti-TNF activity was developed for IBD therapy 14. However, the antibody requires a fairly high dose to achieve remission of symptoms as most of the antibodies may degrade in the GI tract. Several barriers, such as digestive enzymes in the GI tract and poor membrane permeability, make the oral delivery of antibody a great challenge 15.

Therefore, there is a great need for oral delivery systems of antibodies in order to improve the efficacy and reduce the side effects in the treatment of IBD 16, 17. Oral drug delivery systems for various macromolecules have been studied recently 18, 19. Nanoparticulate drug delivery systems are of particular interest in the treatment of colitis and colitis-associated cancer due to their small size and versatile surface chemistry 20-27. The increased permeability of epithelium allows the nanoparticles to accumulate in the inflamed intestine through oral delivery 28, 29. In previous work, we developed polyphenol-poloxamer self-assembled supramolecular nanoparticles for oral delivery in IBD therapy 30. Natural polyphenols such as tannic acid (TA) and epigallocatechin gallate (EGCG) are rich in galloyl and catechol groups that form hydrogen bonds and hydrophobic interactions with various proteins and peptides 31, 32. Antibodies, such as herceptin (trastuzumab) and anti-PD-L1 blocking antibody (aPDL1), can be delivered to tumor *in vivo* by EGCG-based nanoplatform with significant improvement efficacy 33, 34. In addition to protein, polymers such as poly(ethylene glycol) (PEG), poly(N-vinyl pyrrolidone) (PVP), poly(diallyldimethylammonium chloride) (PDDA) and poly(sodium 4-styrene sulfonate) (PSS) can also be captured by TA through hydrogen bonding 35. Therefore, biocompatible and versatile polyphenol-based biomaterials have attracted increasing attention 36-41.

Herein, we demonstrate an approach to deliver antibody orally by using hydrogen bonding supramolecular nanoparticles assembled with TA and 1,2-distearoyl-sn-glycero-3-phosphoethanolamine-N-[methoxy(polyethylene glycol)-2000] (DSPE-PEG2k). Infliximab, a chimeric IgG molecule consisting of a human Fab' fragment combined with a murine Fc fragment, was used as a model antibody therapeutic. It has been demonstrated to have clinical efficacy in both inducing and maintaining remission in Crohn's disease (CD) and ulcerative colitis (UC) 42. And it has also been showed that infliximab has good affinity for both human-TNF-α and murine-TNF-α 43. Infliximab can be protected by nanoparticles in the GI tract without degradation and targeting to the site of intestinal inflammation. When encountered the high reactive oxygen species (ROS) at the site of inflammation, the nanoparticles are degraded and release antibodies for TNF-α inhibition. Therefore, a significantly enhanced therapeutic efficacy can be achieved compared to the free antibody through orally delivery. The average weight, colon length, and inflammatory factors in colon and serum of colitis mice after the treatment of novel formulation of anti-TNF-α antibodies even reached the similar level to healthy controls.

## Results and Discussion

### Aqueous self-assembly of nanoparticles by TA and PEG containing polymers

**Figure 1:**
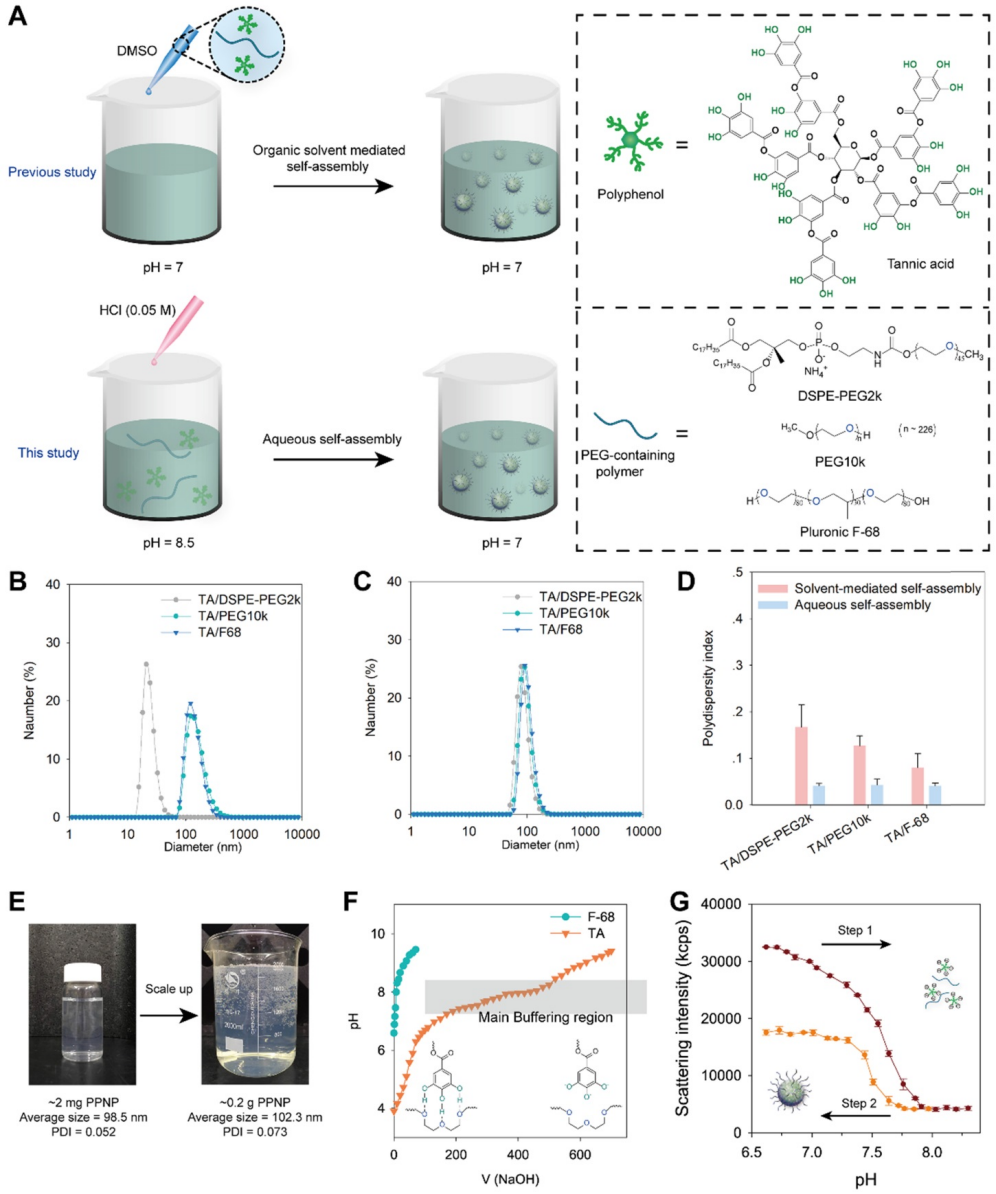
** (A)** Schematic illustration of the preparation process of TA and PEG containing polymers assembled nanoparticles through organic solvent-mediated self-assembly (in previous study) and aqueous self-assembly (in this study). The size distribution of nanoparticles fabricated by organic solvent-mediated self-assembly **(B)** and aqueous self-assembly **(C)** determined by DLS. **(D)** Polydispersity index comparison of nanoparticles fabricated by organic solvent-mediated self-assembly and aqueous self-assembly. **(E)** Scale-up of nanoparticles fabrication by TA and DSPE-PEG2k from 2 micrograms to 0.2 gram. The volume changed from 20 mL to 2 L. **(F)** pH titration of TA and F-68 by 0.1 N NaOH. **(G)** Scattering intensity of nanoparticles with pH change from 6.6 to 8.3 and then reverse from 8.3 to 6.6.

In previous work, we fabricated polyphenol-poloxamer self-assembled nanoparticles through organic solvent-mediated assembly 30, 44. Polyphenol and PEG-containing polymers were first mixed in organic solvent and then added to PBS buffer at pH 7 to obtain polyphenol-PEG-containing polymers self-assembled nanoparticles (PPNP) (Figure 1A). However, owing to the toxic potential of organic solvents, the presence of organic solvent residues is a great challenge to the scale-up of nanomedicine 45. Liposome, one of the most successful nanomedicine in clinical translation, require removal and assessment of organic solvent residues during the fabrication process 46. TA have five galloyl and catechol groups that can form hydrogen bonds with macromolecules such as polymers and proteins. The protonation and deprotonation of galloyl and catechol groups can be easily achieved by adjusting the pH. Herein, inspired by dynamic protonation and deprotonation of galloyl and catechol groups tuned with pH, we demonstrated aqueous self-assembly of TA and PEG-containing polymers including DSPE-PEG2k, PEG10k and Pluronic F68. As shown in Figure 1A, TA and a PEG-containing polymer were added to a PBS buffer at pH 8.5, and then the pH was adjusted to 7.0 *via* adding 0.05 M HCl dropwise. The hydrodynamic sizes of three types of nanoparticles prepared by aqueous self-assembly or organic solvent-mediated self-assembly were determined by dynamic light scattering (DLS). As shown in Figure 1B, the nanoparticles fabricated by TA and DSPE-PEG2k through organic solvent-mediated self-assembly have a unique hydrodynamic size of 24 nm while the others are approximately 142 nm. All the three types of nanoparticles fabricated through aqueous self-assembly with different components are approximately 100 nm (Figure 1C). The polydispersity index (PDI) of the three types of nanoparticles prepared by aqueous self-assembly is extremely low (less than 0.1), which is obviously lower than that of nanoparticles prepared by organic solvent-mediated self-assembly. The fabrication process was also scaled up from ~ 2 mg to ~ 0.2 g weight of nanoparticles. The size and PDI of the nanoparticles are almost unchanged (Figure 1E). The simple preparation process, no organic solvent toxicity, and highly feasible scale-up of this nanoparticle demonstrate the great potential for commercialization.

To investigate the mechanism of aqueous self-assembly of TA and PEG-containing polymers, we measured the pH buffering capacity of TA. F68 was also measured as control which has no functional groups can be protonated. As shown in Figure 1F, TA has a much strong pH buffering capacity than F68. And the main pH buffering region is from 6.8 to 8 which means the majority of the phenolic group in TA have been deprotonated during this pH change. Furthermore, the reversible assembly and disassembly of TA and F-68 by pH change was investigated. The nanoparticles were disassembled when pH changed from 6.6 to 8.3 (Figure 1G). And when the pH reversed back from 8.3 to 6.6, the nanoparticles formed again. When the pH rises from 6.6 to 8.3, part of the galloyl and catechol moieties of tannic acid are oxidized due to the rapid auto-oxidation of catechol at alkaline pH 47. Therefore, when the pH is reduced back from 8.3 to 6.6, the number of self-assembled nanoparticles formed decreases as the unoxidized tannic acid concentration decreases. The pH titration results in Figure S1 showed that the PPNP could aggregate into large size nanoparticle when pH less than 4. And when pH increased to 8, PPNP will degrade. These results reveal that the aqueous self-assembly approach is pH-dependent. Thus, we speculate that thanks to the slow progress of aqueous self-assembly by pH titration, the nearly monodisperse self-assembled nanoparticles can be obtained even on large scale.

### Encapsulation of protein in PPNP

**Figure 2:**
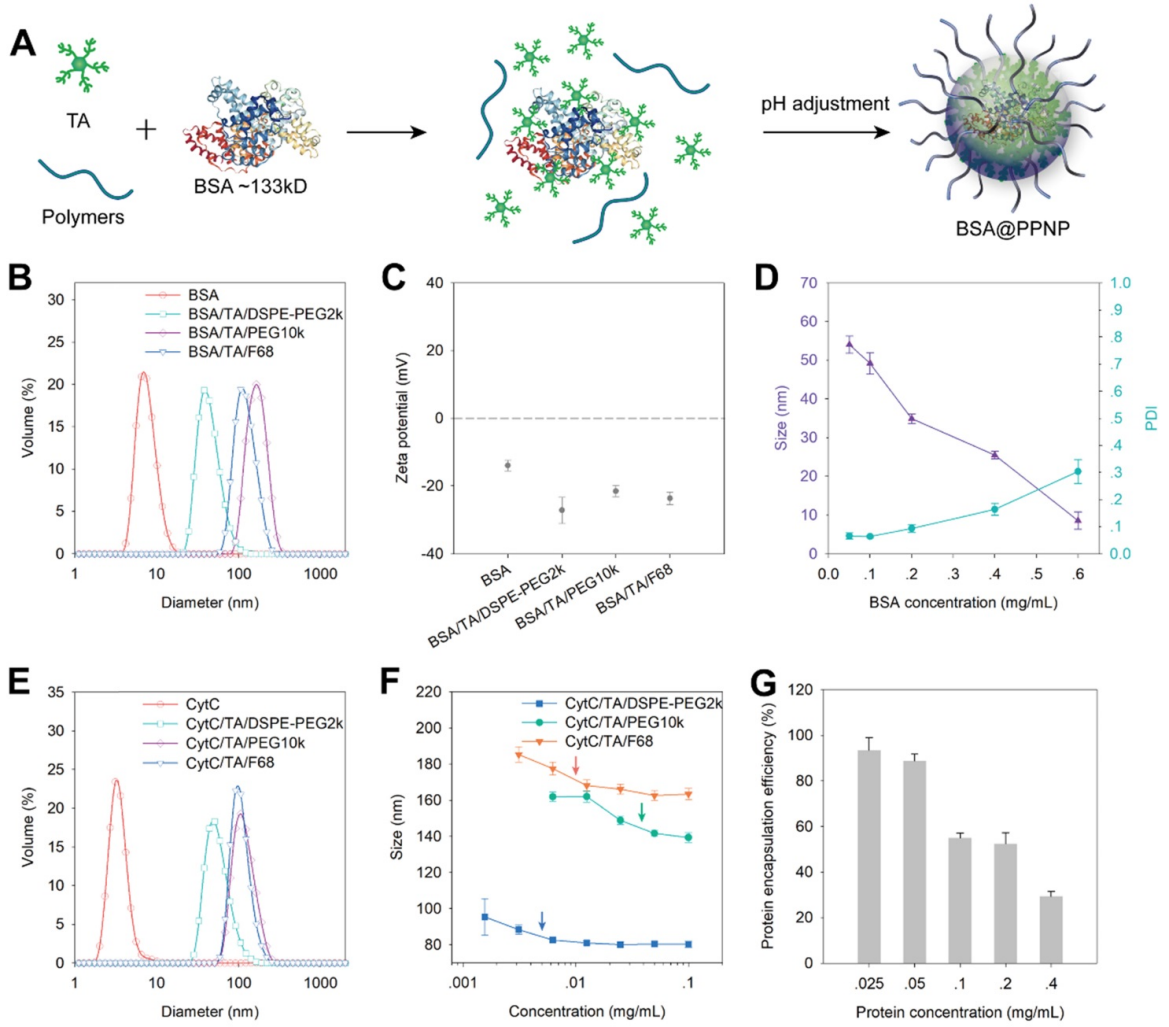
(**A**) The model protein BSA were loaded into PPNP by assembled with TA and PEG containing polymers. The protein first formed complexes with TA through hydrogen bonding and ionic bonding. After pH adjustment from basic to neutral, the protein was encapsulated into supramolecular nanoparticles. (**B**) Size distribution and (**C**) zeta potential of BSA, the nanoparticles formed by BSA, TA with DSPE-PEG2k, PEG10k, and F68 which was determined by DLS. (n = 3) (**D**) Average size and PDI of BSA@PPNP fabricated by TA, DSPE-PEG2k and various concentrations of BSA. (n = 3) (**E**) The size distribution of CytC, the nanoparticles formed by CytC, TA with DSPE-PEG2k, PEG10k, and F68. (**F**) The average size of nanoparticles formed by CytC, TA and polymers at different concentration reflected by DLS. (n = 3) (**G**) The protein encapsulation efficiency of CytC@PPNP formed by CytC, TA and DSPE-PEG2k. (n = 3).

We evaluated the protein encapsulation ability of PPNP by using bovine serum albumin (BSA, 66 kDa) and cytochrome c (CytC, 12 kDa) as model proteins. Owing to the protein binding ability of TA, it can form complexes with BSA in aqueous solution first. And then the BSA loaded nanoparticles (BSA@PPNP) can be obtained by pH adjustment (Figure 2A). DLS results show that all the three types of nanoparticles can form uniform nanoparticles with the different size around 100 nm after loading BSA (Figure 2B). The zeta potential of the nanoparticles and BSA was also tested. The zeta potentials of all the three types of nanoparticle are less than -20 mV, which is beneficial for targeting the positively charged surface of the inflamed colon 29. With the increase of BSA concentration during BSA@PPNP preparation, the size of nanoparticles decreased and PDI increased (Figure 2D). This may be due to the blocking of the galloyl and catechol groups of the tannic acid by proteins, resulting in less interaction between TA and PEG. CytC was loaded by the three types of nanoparticles with results similar to BSA (Figure 2E). We also evaluated the critical aggregation concentration (CAC) of these three nanoparticles with CytC loaded by DLS. As shown in Figure 2F, the nanoparticles obtained by self-assembled TA, DSPE-PEG2k and CytC exhibit the smallest CAC which is less than 7 mg/L. This may be attributed to the hydrophobic interaction between DSPE chains that enhanced the stability of this nanoparticle. As shown in Figure S2, the CAC of INF@PPNP is also less than 7 mg/L, which is similar to the CAC of CytC@PPNP. The results indicate that the protein type may have no obvious effect on the CAC of nanoparticles. Thus, this kind of nanoparticle with better stability was studied further. The protein loading ability was measured by ultrafiltration after nanoparticle preparation with a series of protein concentrations. The results in Figure 2G show that as the protein concentration less than 0.05 mg/mL, the loading efficiency was greater than 90%. The protein loading efficiency decreases with the increase of protein concentration, which is attributed to the decreased interaction between TA and the other two components. We compared the protein encapsulation efficiency of PPNP with CytC (12 kDa), BSA (66 kDa), and INF (149 kDa) at a protein concentration of 0.05 mg/mL (Figure S3). It can be found that all the three types of protein with a wide range of molecular weight reached encapsulation efficiencies more than 90%. These results indicate that the molecular weight of protein will not obviously affect the efficiency of protein.

The stability of INF@PPNP in saline (37 °C, pH 7.4) was also studied (Figure S4). It was showed that the size of INF@PPNP increased slightly in first 24 h and remained stable for the next 48 h, while the PDI remained approximately 0.1 for 72 h. The results indicate that INF@PPNP has high colloidal stability.

In order to study the effect of protein loading on protein structure and activity, we performed circular dichroism spectroscopy and enzyme activity testing. Circular dichroism results in Figure S5 showed that protein secondary structures have a little change after protein loading process. However, protein secondary structures can be recovered after the protein released from the PPNP nanoparticles. The model enzymes α-amylase and xanthine oxidase loaded in nanoparticle did not affect the enzyme activity after released from PPNP nanoparticles (Figure S6). These results indicated that both the protein structure and enzyme activity will not change obviously if they can be released from PPNP nanoparticles. We also compared anti-TNF-α ability of free INF, INF@PPNP and INF released from SIG treated INF@PPNP. The results in Figure S7 showed that the INF released from SIG treated INF@PPNP maintained comparable anti-TNF-α capacity with free INF, indicating that PPNP has a protein protection ability against acid environment in stomach. All these results suggest that the advantages of PPNP would facilitate the oral delivery of therapeutic antibodies.

### The responsive behavior of protein loaded nanoparticles

**Figure 3:**
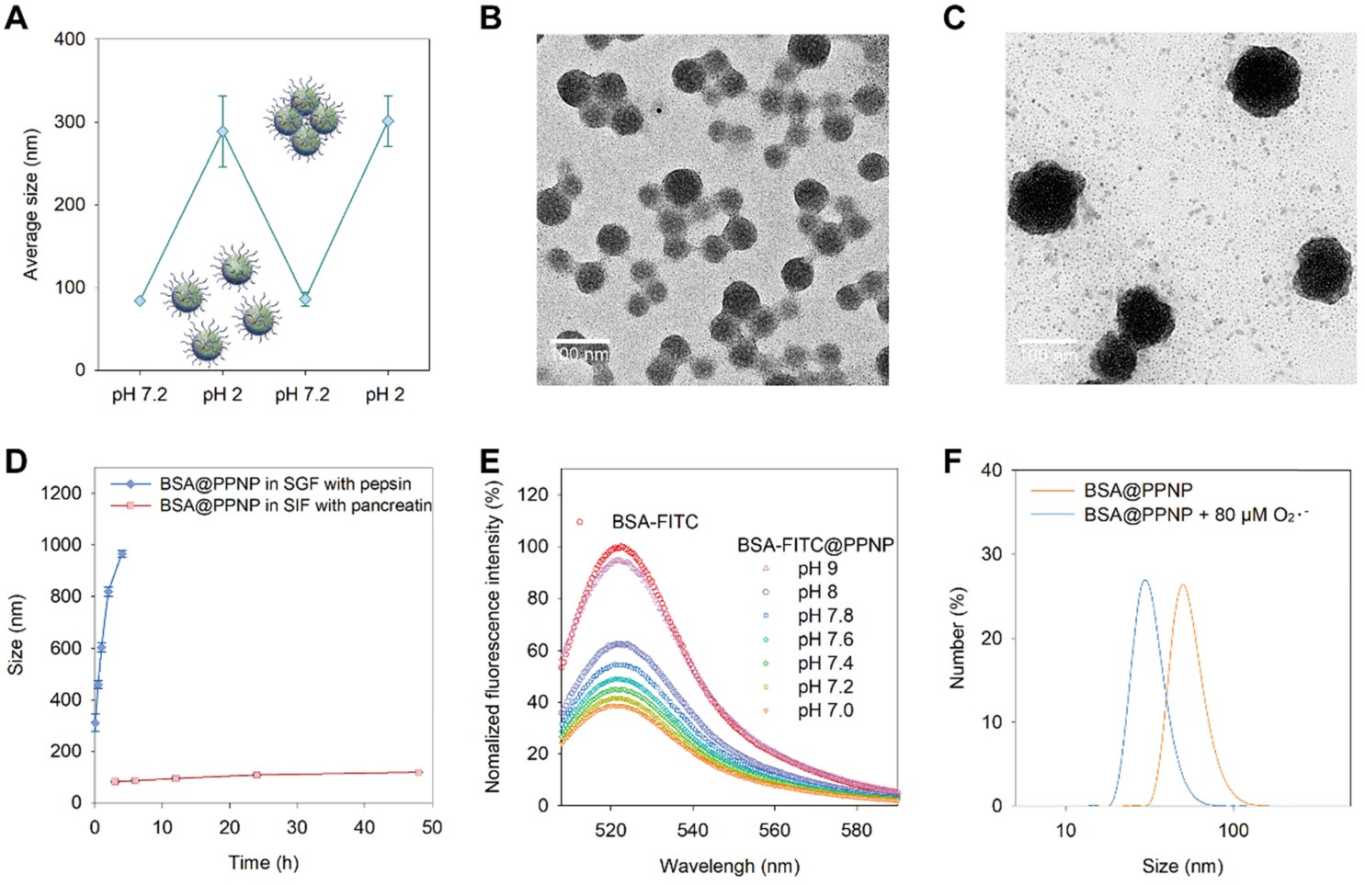
** Stimuli-responsive behavior of BSA@PPNP.** (**A**) Size change of BSA@PPNP in pH 7.2 and 2 cycles. (n = 3) TEM of BSA@PPNP in pH 7.2 (**B**) and pH 2 (**C**). Scale bar is 100 nm. (**D**) Size change of BSA@PPNP at a different time in SGF with pepsin or SIF with trypsin. (n = 3) (**E**) Fluorescence spectra of BSA-FITC@PPNP at different pH value. (**F**) The size distribution of BSA@PPNP with or without 80 µM O_2_•^-^.

The dynamic pH environment of the GI tract is between 1 and 7.9. As drugs entered the stomach after oral administration, it will meet the acid environment. And when it went through the stomach and reached intestine, the pH of the environment will turn to neutral or slightly alkaline. We speculate the protein-loaded nanoparticles have a stimuli-responsive behavior in dynamic pH environment of the GI tract based on the pH-dependent dynamic hydrogen bonding of PPNP. As shown in Figure 3(A), BSA@PPNP aggregated into large particles which have a hydrodynamic size of more than 300 nm when the pH changed from 7.2 to 2. Interestingly, when the pH value reversed from 2 to 7.2, the hydrodynamic size of BSA@PPNP could also be reversed back to less than 100 nm. The transmission electron microscopy (TEM) images in Fig 3(B and C) also revealed the pH responsive behavior of BSA@PPNP. We also tested the size change behavior of BSA@PPNP in simulated gastric fluid (SGF) and simulated intestinal fluid (SIF). As shown in Figure 3D, the hydrodynamic size of BSA@PPNP in SGF with pepsin increased from 100 nm to 1 μm in 2 hours while it changed not obviously in SIF with pancreatin. To stimulate the process from stomach to intestine, INF@PPNP was first added into SGF and neutralized by SIF. The size change results in Figure S8 obtained by DLS suggest that the INF@PPNP aggregated in acidic environment of stomach but invents to small-sized nanoparticles (less than 100 nm) in the intestine. All these results indicate that protein-loaded PPNP aggregates into larger size nanoparticles in the stomach, but reverts into smaller nanoparticles when it reached the intestine.

To investigate the effect of pH change between 7 and 8 on BSA@PPNP, the BSA-FITC was also loaded in PPNP. Owing to the aggregation-caused quenching effect, the fluorescence of BSA-FITC was decreased obviously after loading in nanoparticle compared with free BSA-FITC (Figure 3E). When the BSA-FITC@PPNP was dissociated and BSA-FITC was released, the fluorescence can be recovered. We can find that the fluorescence can be almost fully recovered at pH 9. But between pH 7.0 and 7.8, only a small part of quenched fluorescence can be recovered. These results suggested that protein-loaded nanoparticle could be quite stable in the pH environment of the intestine. It was reported that reactive oxygen species (ROS) concentration increased 10- to 100-fold in the mucosa of ulcerative colitis and Crohn's disease 48. Thus, we tested the size change of BSA@PPNP with the stimulation of reactive oxygen species. The DLS results in Figure 3F showed that the hydrodynamic size of BSA@PPNP decreased from approximately 70 nm to less than 30 nm after incubation with 80 μM O_2_•^-^. Due to the pH decreasing in colons with IBD, we also did the ROS-responsive decomposing test in SIF at pH 5.5 (Figure S9). It showed similar results with that in SIF at pH 7.2. These results indicate that protein-loaded nanoparticle may dissociate with the stimulation of reactive oxygen species in inflamed colon tissue. This can be explained by that the oxidation of galloyl group affects the assembly stability of PPNP. The phenolic hydroxyl groups on galloyl groups of tannic acid are easily oxidized to ketone groups by ROS, thus destroying the original hydrogen bonds between tannic acid and PEG chains.

### Inflamed colon targeting of nanoparticles

On the basis of the dynamic size of protein-loaded PPNP in the GI tract, we next checked whether it would contribute to the inflamed colon targeting of INF@PPNP. We investigated the interactions of INF@PPNP with mucin and transferrin coated surface to simulate the healthy and inflamed epithelium, respectively (Figure S10). The transferrin coated surface retained much more Cy5.5-labeled INF@PPNP than uncoated or mucin coated surface, which indicates that it tends to adhere to inflamed colons. The negative surface charge of the INF@PPNP facilitate its adhesion to the positively charged inflamed colon epithelium, which is consistent with the literature.

**Figure 4:**
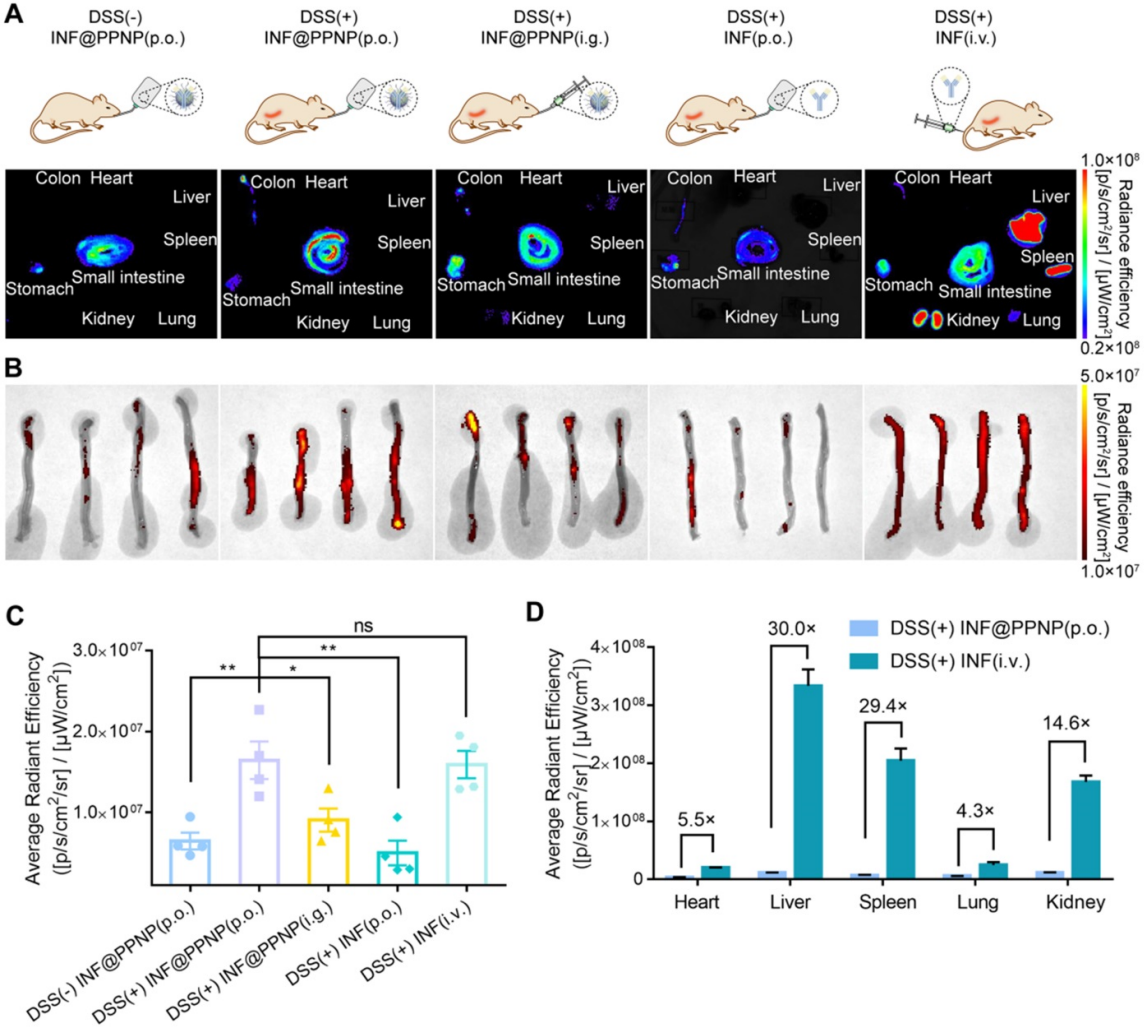
** Biodistribution of antibody *via* various administration routes.** (**A**) Representative fluorescence images of tissues and organs (including heart, liver, spleen, lung, kidney, stomach, small intestine, and colon) in five groups at 24 h post-administration. Group (I): Healthy mice treated with INF@PPNP (p.o.). The DSS induced colitis mice were treated with INF@PPNP (p.o.) (II), concentrated INF@PPNP (i.g.) (III), INF (p.o.) (IV), and INF (i.v.) (V). The INF was labeled with Cy5.5. (**B**) The fluorescence images of colons in five groups. (**C**) Average radiant efficiency of colon tissues in five groups. (**D**) Average radiant efficiency of main organs including heart, liver, spleen, lung and kidney in Group (II) and (V). Values are expressed as the means ± SEM (n = 4). *p < 0.05, **p < 0.01, ***p < 0.001, n.s. p ≥ 0.05 analyzed by one-way ANOVA with Tukey's post hoc test performed on the same 4 animals per groups.

*In vivo* study was also performed. There were five study groups: four with DSS induced colitis and one healthy control. Colitis mice were treated with INF@PPNP or INF *via* various administration route including *per os* (p.o.), intragastric (i.g.), and intravenous (i.v.). Healthy controls were treated with INF@PPNP (p.o.). The concentration of INF in p.o. administration groups are much lower than i.g. and i.v. administration groups. As the INF was labelled with NIF fluorescence dye Cy5.5, the organ and tissues were analyzed by IVIS at 24 h post-treatment. As shown in Figure 4 (A to C), orally administrated INF@PPNP increased the concentration of INF in inflamed colons significantly compared to that of healthy controls. And it was also higher than that of i.g. administration group, but close to that of the i.v. administration of free INF. This may be due to the fact that INF@PPNP can aggregate into larger nanoparticles at higher concentration in i.g. administration group. Thus, more INF remained in the stomach of i.g. administration group than p.o. administration. Furthermore, it can be found that, in the orally administration groups (p.o. and i.g.), INF mainly distributed in the GI tract, such as the stomach, small intestine, and colon (Figure 4A and D). However, the INF mainly distributed in liver, spleen and kidney after intravenous administration. The amount of INF in heart, liver, spleen, lung and kidney in i.v. administration group increase to approximately 5.5, 30, 29.4, 4.4 and 14.6-fold compared with p.o. administration group. These results demonstrate that p.o. administration of INF@PPNP can remarkably reduce systemic exposure of INF while maintaining high drug level in the inflamed colon.

The time-course analysis of INF@PPNP biodistribution in DSS-induced mice was also studied. The mice were treated orally with Cy5.5-INF@PPNP for 12 h first and then replaced by water. The mice were sacrificed at 0, 4, 12, and 24 h after the water replacement. As shown in Figure S11, there is no obvious accumulation can be found in heart, liver, spleen, lung and kidney. And after the replacement of Cy5.5-INF@PPNP by water, the fluorescence in stomach, small intestine and colon began to decrease. However, the fluorescence in liver increased only a little in the next 24 h. In colon, the fluorescence maintained a relative high level for at least 4 hours and decreased obviously at 12 h and 24 h after replacement.

### Treatment of DSS-induced colitis

**Figure 5:**
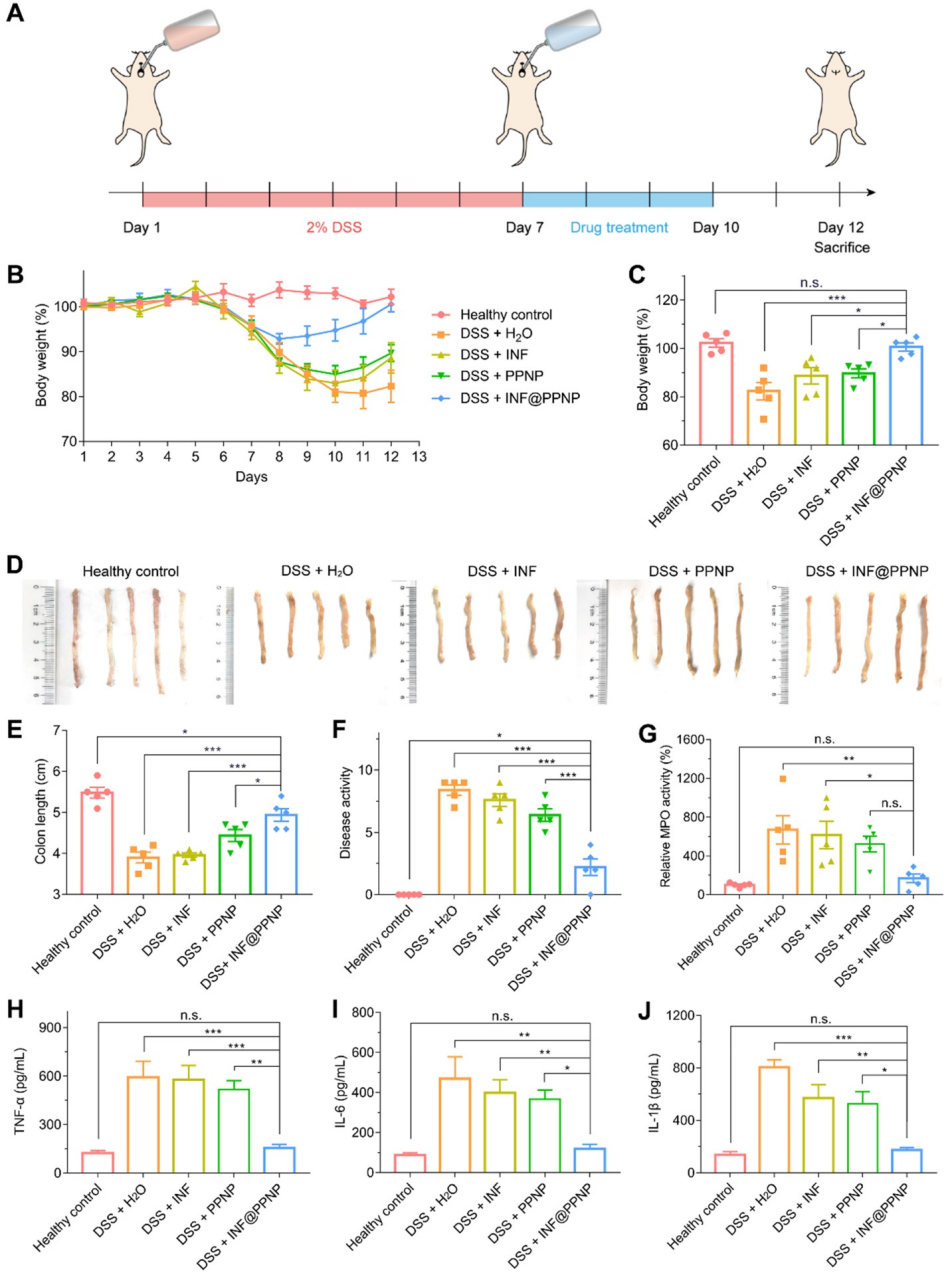
(**A**) Schematic illustration of DSS induced colitis in C57/BL6 mice and treatment schedule. (**B**) Bodyweight of mice treated with water, INF, PPNP and INF@PPNP after 2% DSS induced colitis. The healthy control mice were not treated during the therapeutic period. (**C**) Bodyweight on day 12 normalized to that on day 1. (**D**) Photos of the mice colons and (E) measurement of colon length from various treatment groups. (**F**) Disease activity index on day 12. (**G**) Relative MPO activity of mice colon tissues after treatment. (H) TNF-α (I) IL-6 (J) IL-1β levels of mice colon tissues determined by ELISA. Values are expressed as the means ± SEM (n = 5). *p < 0.05, **p < 0.01, ***p < 0.001, n.s. p ≥ 0.05 analyzed by one-way ANOVA with Tukey's post hoc test performed on the same 5 animals per groups.

We then examined the *in vivo* treatment efficacy of IFN@PPNP in DSS-induced colitis mice. Treatment is performed immediately after the colitis induction period. There were five study groups: four with DSS induced colitis and one healthy control. INF, PPNP and INF@PPNP were given to colitis mice as drinking water for 3 days. The INF concentration of free INF and INF@PPNP was set at 40 μg/mL to ensure 10 mg/kg of drug per day, since the mice drank an average of 5 mL water per day. Healthy mice and untreated mice were served as negative and positive controls. All mice were sacrificed on day 12 for further examination (Figure 5A). Bodyweight loss is an important indication for colitis models. As shown in Figure 5 (B and C), weight loss is recovered significantly in INF@PPNP group compared with H_2_O, INF, and PPNP groups. The bodyweight of mice in INF@PPNP group is even close to the healthy control group on day 12 which demonstrated the high efficacy of INF@PPNP. The colon length, disease activity and myeloperoxidase (MPO) activity were also analyzed for treatment efficacy evaluation further (Figure 5, D to G). We observed the colon length in INF@PPNP group is significantly longer than that of all the other experiment groups, except for healthy control. Both disease activity and MPO activity of mice in INF@PPNP group were reduced most significantly among all the experiment groups. The treatment efficacy of PPNP was also observed here, which may owe to the anti-inflammation ability of tannic acid 49. However, the free INF showed no efficacy through orally delivery which may due to the degradation by digestive enzymes in the GI tract.

**Figure 6:**
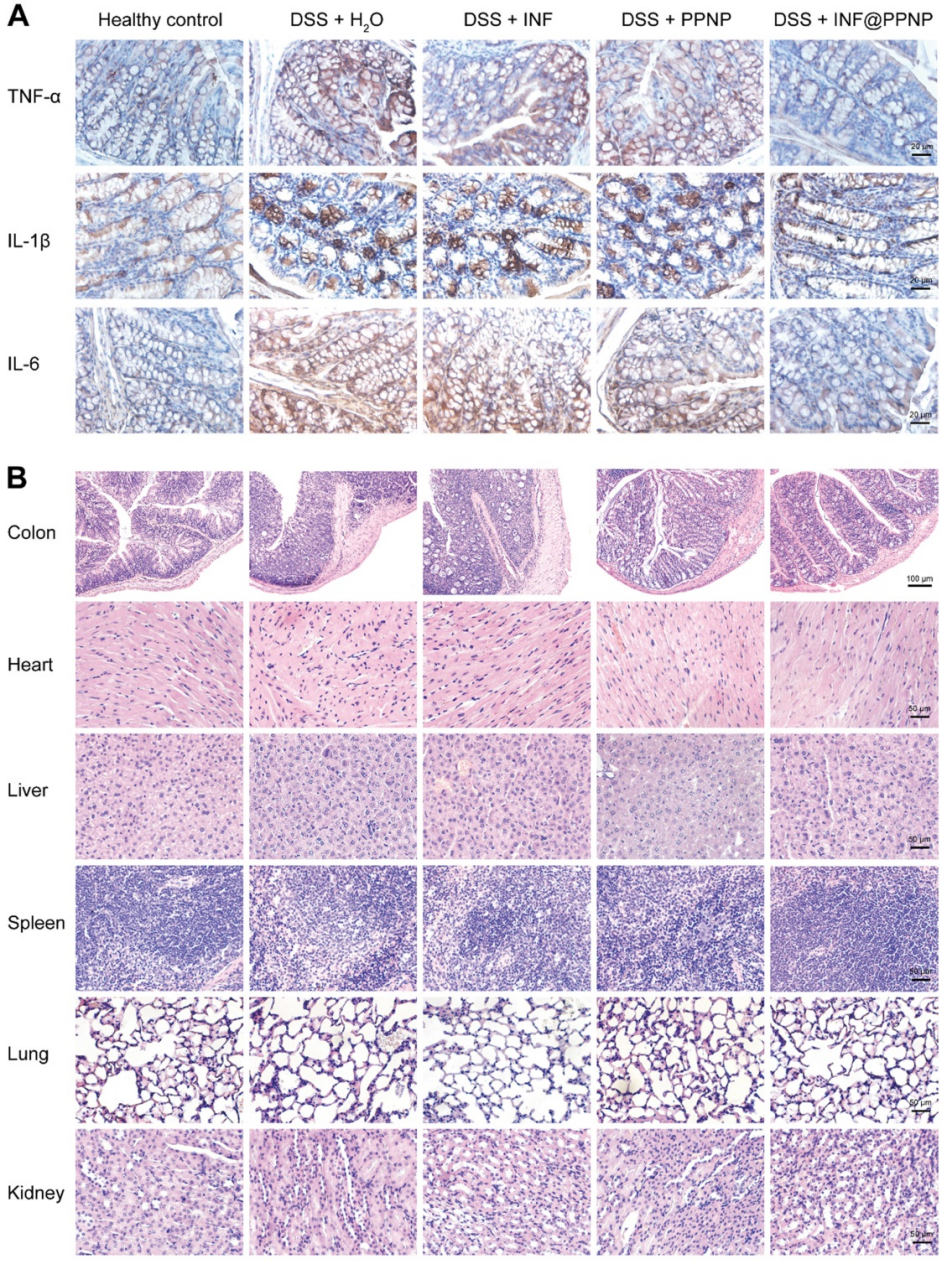
(**A**) Immunohistochemical staining for TNF-α, IL-1β, and IL-6 in colon tissues of different groups. (**B**) Representative H&E-stained histological sections of colon, heart, liver, spleen, lung, and kidney of different groups.

TNF-α, IL-1β and IL-6 are proinflammatory cytokines produced by activated macrophages during IBD 50. Thus, we tested the TNF-α, IL-1β and IL-6 level in colonic tissue by enzyme linked immunosorbent assay (ELISA). As shown in Figure 5 (H to J), the TNF-α, IL-1β and IL-6 level of INF@PPNP group was reduced to the same level of healthy control, which is significantly lower than that of H_2_O, INF, and PPNP groups. Immunohistochemistry results in Figure 6A also are consistent with the ELISA results.

Furthermore, the histological examination by hematoxylin and eosin (H&E) were performed to evaluate the treatment efficacy. Results in Figure 6B showed well-defined crypt structures and relatively low levels of neutrophil invasion was found in INF@PPNP group compared with H_2_O, INF, and PPNP groups.

Further we compared the efficacy of IFN@PPNP (p.o.) with free IFN administered intravenously *in vivo*. As shown in Figure S14, body weight and colon length measurements suggest that IFN@PPNP (p.o.) achieved similar therapeutic efficacy with free IFN administered intravenously. The inflammatory factors in serum such as TNF-α, IL-1β and IL-6, also confirmed that.

And there was no significant pathological change in the main organs (liver, kidney, spleen, lung and heart) of the mice in each group (Figure 6B), indicating that the systemic toxicity caused during INF@PPNP treatment was minimal. The cytotoxicity of PPNP and INF@PPNP was also tested in HT29 cells (human colon carcinoma cells). No obvious cytotoxicity of PPNP and INF@PPNP can be found at concentrations less than 0.1 mg/mL (Figure S12). Further to investigate the systemic biocompatibility of the blank materials PPNP, blood from normal mice and PPNP treated mice was collected one week after treatment for analysis of hematological parameters and liver function biomarkers. The results in Figure S13 showed that no significant difference of white blood cells (WBC), red blood cells (RBC), hemoglobin (HGB), platelet (PLT), aspartate aminotransferase (AST) and alanine aminotransferase (ALT) levels between healthy mice without treatment and INF@PPNP treated mice. Hematological parameters and liver function analysis indicate that the blank material PPNP is biocompatible with oral treatment.

**Figure 7:**
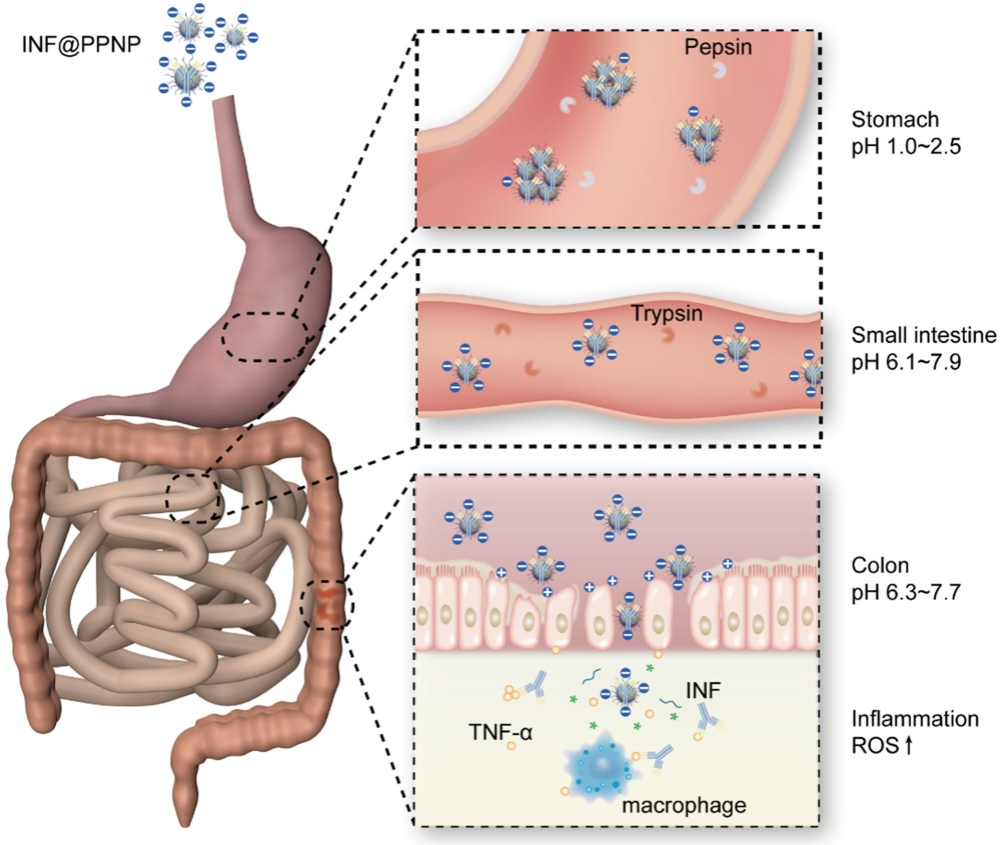
Schematic depiction of colitis treatment by INF@PPNP. With oral treatment into mice, the INF@PPNP aggregate into large size nanoparticles (>300 nm) at acid pH value in the stomach. The aggregated nanoparticles can protect the INF from degradation by pepsin. After the aggregated nanoparticles transferred into the small intestine, it can be reversed into small size nanoparticles (~100 nm) at neutral pH value. And the nanoparticles can be captured by positively charged proteins of inflamed colon surface and penetrated into colon tissue with high epithelial permeability. The INF can be released by degradation of the nanoparticles by high ROS level in the inflammation tissue.

All these results demonstrated the strong treatment efficacy of INF@PPNP for oral delivery in colitis treatment. As illustrated in Figure 7, the INF@PPNP first aggregated into large size nanoparticles in the stomach. And when it transferred into the intestine, it can be reversed back to be small size nanoparticles (~100 nm) at neutral pH value. And the negatively charged nanoparticles can be captured by positively charged proteins of inflamed colon surface and penetrated into colon tissue with high epithelial permeability. The assembled nanoparticles can be disassociated by high ROS level in the inflamed colon mucosa. Thus the proinflammatory cytokine TNF-α can be inhibited by released INF.

Although there have been many studies on oral delivery of drugs for IBD therapy, few of them were performed to deliver protein drug. Cai *et al.* showed that oral delivery of TGF-1 gene-modified DCs-derived exosomes can specifically interact with T-cell subsets to induce CD4+Foxp3+Tregs and reduce the proportion of Th17 cells at the sites of inflammation, thereby inhibiting the development of DSS-induced colitis in mice 51. To the best of our knowledge, our study is the first study of orally administered monoclonal antibodies for IBD treatment.

## Conclusions

In summary, we fabricated tannic acid and poly(ethylene glycol) containing polymers self-assembled supramolecular nanoparticle which could load various proteins. The aqueous self-assembly of this nanoparticle allowed it to be easily produced in large scale and organic solvent-free in products. The dynamic hydrogen bonding-mediated assembly allowed the nanoparticle to be size tunable in the GI tract. The oral administration of antibody loaded nanoparticle increased the antibody accumulation in the inflamed colon compared with the free antibody. Furthermore, it also reduced systemic exposure compared with i.v. administration. This improved formulation of orally administered therapeutic antibodies was effective in colitis mice, which also provides an excellent platform for the oral administration of antibodies for various GI tract disease.

## Experimental Section

### Materials

Tannic acid, PEG10k, F-68, KO_2_, α-amylase, xanthine oxidase, BSA, fluorescein 5(6)-isothiocyanate, pepsin (from porcine gastric mucosa) and pancreatin (from porcine pancreas) were bought from Sigma-Aldrich. DSPE-PEG2k was obtained from Advanced Vehicle Technology Pharmaceutical Co., Ltd (Shanghai, China). Cy5.5-NHS were purchased from (ApexBio Technology, USA). Cytochrome C was obtained from Sangon Biotech (Shanghai, China). Infliximab was purchased from BioChemPartner (Shanghai, China). Dextran sodium sulfate (molecular weight: 36,000-50,000 Da) were purchased from MP Biomedicals Inc. (USA).

### Preparation of nanoparticles

For organic solvent-mediated self-assembly, TA, DSPE-PEG2k, PEG10k, and F68 were first dissolved in DMSO at a concentration of 20 mg/mL. Afterwards, TA and one of the three polymers were mixed at a weight ratio of 1:1. Finally, the mixture in DMSO was added dropwise into 10 mM PBS at a final concentration of 0.1 mg/mL to obtain the self-assembled nanoparticles. For aqueous self-assembly, TA, DSPE-PEG2k, PEG10k, and F68 were first dissolved in DI water at a concentration of 10 mg/mL. Then, one of the three polymers was added to 10 mM PBS (pH 8.5-9.0) at a final concentration of 0.05 mg/mL. Finally, the same amount of TA was added to the solution and the pH was adjusted to approximately 7 using 0.01M HCl. Hydrodynamic size, zeta potential, scattering intensity and pH titration measurements by DLS were performed with a Zetasizer Nano ZSE equipped with MPT-2 autotitrator (Malvern Instruments, Ltd.).

### Preparation of protein-loaded nanoparticles

To prepare the protein-loaded nanoparticle, an aqueous protein solution (5 mg/mL in PBS) was mixed with an aqueous TA solutions (5 mg/mL in DI water) at different weight ratios. The mixture was added to 10 mM PBS pH 8.5-9.0) with TA concentration of 0.1 mg/mL. After that, DSPE-PEG2k, PEG10k, or F68 aqueous solutions (5 mg/mL) were added to the mixture at a final concentration of 0.1 mg/mL. Finally, the pH was adjusted to 7 using 0.01 M HCl to obtain the protein-loaded nanoparticles. Protein-loaded PPNP was transferred to a dialysis bag (MWCO 14 kD) and dialyzed against 0.5 L DI water six times under magnetic stirring for 8 hours. For infliximab loading, the antibody was purified with PD-10 desalting columns (GE Healthcare Life Sciences) before nanoparticle fabrication. The other steps were just the same as for other proteins. To determine the protein encapsulation efficiency, CytC loaded nanoparticle solutions were centrifuged in Millipore ultrafiltration device (MCWO is 100 kD) at 5000 rpm for 10 min. The filtered solutions were collected for protein quantification assay. The protein concentration was determined by bicinchoninic acid (BCA) protein assay kit (Beyotime, Shanghai, China) according to its protocol.

### The responsive behavior of protein loaded nanoparticles

The pH-responsive study was performed by adjusting pH using Zetasizer Nano ZSE equipped with MPT-2 autotitrator. For TEM analysis, the as-prepared BSA@PPNP was adjusted to pH 2 or 7.2 by 0.01 M HCl or NaOH. One drop of BSA@PPNP at pH 2 or 7.2 were collected and deposited on 400 mesh carbon-coated copper grids, respectively. Excess liquid was removed using filter paper or evaporated into air in a couple of minutes. TEM analysis was performed on JEM-2100 transmission electron microscopy (JEOL, Japan) operating at an acceleration voltage of 200 kV. The size change of BSA@PPNP in simulated gastric fluid (SGF) and simulated intestinal fluid (SIF) was also tested by DLS after incubation over a series of the set time. Simulated gastric fluid (SGF) was prepared with pepsin (1%), pH 1.2, which consists of 2.0 g/L NaCl and 7 mL/L of concentrated HCl. Simulated intestinal fluid (SIF) was prepared with pancreatin (10%) pH 7.2, which consists of 50 mL NaH_2_PO_4_ 0.1 M and 42.5mL NaOH 0.1 M for 1 L of solution. SGF and SIF were filtered with a 0.22 μM Millipore filtration membrane after preparation. The O_2_•^-^ was incubated with BSA@PPNP to study the ROS responsive behavior. The O_2_•^-^ was generated by the injection of KO_2_ in dimethyl sulfoxide (DMSO) into the phosphate buffer solution. Briefly, A 10 mM stock solution of KO_2_ was prepared by adding 1.41 ml of DMSO to 1.0 mg KO_2_ and sonicating for 5 min. The hydrodynamic size of BSA@PPNP was determined by DLS before and after incubation with O_2_•^-^ for 5 min. The fluorescence spectra of FITC-BSA and FITC-BSA@PPNP with various pH were detected by a fluorescence spectrophotometer (LS55, PerkinElmer).

### Colitis models

All animal experiments were conducted following the National Institutes of Health guidelines for the care and use of laboratory animals and approved by the Institutional Animal Care and Ethics Committee of Jiangsu Institute of Nuclear Medicine (Wuxi, China). Colitis was induced by feeding 2% DSS to female C57BL/6 mice (SLAC Laboratory Animal Co., Ltd, China, 6 wk, 17-20 g) for 6 d.

### Biodistribution and inflamed colon targeting

After DSS solution feeding finished, the colitis mice were randomly divided into four groups. The colitis and healthy mice were administrated with Cy5.5-INF or Cy5.5-INF@PPNP *via* various administration route, including p.o., i.g., and i.v. The dose of INF was kept the same in all groups which are 10 mg/kg. Group (I): Healthy mice treated with Cy5.5-INF@PPNP (drink) (n=4). The DSS induced colitis mice were treated with Cy5.5-INF@PPNP (drink) (II), concentrated Cy5.5-INF@PPNP (i.g.) (III), Cy5.5-INF (drink) (IV), and Cy5.5-INF (i.v.) (V) (n=4). The INF was labeled with Cy5.5 and purified by dialysis before loading into nanoparticles. The INF concentrations of Cy5.5-IFN (p.o.), Cy5.5-IFN@PPNP (p.o.), Cy5.5-INF (i.v.) and Cy5.5-INF@PPNP (i.g.) are 40, 40, 1000 and 400 μg/mL, respectively. The mice were sacrificed 24 h post-administration. The tissues and organs were collected and observed under the IVIS imaging system (PerkinElmer). The images were analyzed by Living Image® software (Xenogen, CA).

### Colitis treatment

After DSS solution feeding finished, the colitis mice were randomly divided into four groups. The drinking water for colitis mice was replaced by DI water, IFN, PPNP, or IFN@PPNP for 3 days (n=5). The dose of INF was kept the same in all groups which are 10 mg/kg each day. The concentration of INF for IFN and IFN@PPNP is 40 μg/mL. The healthy control mice were not treated during the therapeutic period. The body weight was recorded every day and the severity of colitis in the mice was assessed on day 12. The severity was evaluated by the disease activity index (0-12), including loss of body weight (1, 1-5%; 2, 5-10%; 3, 10-15%; 4, 15-20%), rectal bleeding (0, normal; 1, semi-normal; 2, positive hemoccult; 3, blood traces in stool visible; 4, gross rectal bleeding) and stool consistency (0, normal; 1, semi-normal; 2, loose stool; 3, loose stool that adhered to the anus; 4, liquid stools that adhered to the anus). Colons were collected after the sacrifice of mice and the length was measured.

### MPO activity

Colons were weighed and homogenized in 9 volumes of ice-cold PBS (pH 6.0) containing 0.5% hexadecyltrimethylammonium hydroxide and centrifuged at 12,000 r/min at 4°C for 20 min. 10 mg O-dianisidine hydrochloride was dissolved in 60 ml of 50 mM phosphate-citrate buffer, pH 5.0. Add 12 μl of fresh 30% hydrogen peroxide immediately before to use. 10 μl of supernatants were reacted with H_2_O_2_ containing O-dianisidine hydrochloride solution. Changes in MPO absorbance were measured at 405 nm every 1 min on a microplate reader.

### Enzyme-linked immunosorbent assay (ELISA) methods

Colons were weighed and homogenized in cold PBS (w/v = 1:10) with protease inhibitor cocktail for 20 min. Homogenates were centrifuged at 5000 r/min at 4°C. The supernatants were collected and stored at -80 °C. The TNF-α, IL-1β, and IL-6 cytokines were detected by ELISA kits (Multisciences (Lianke) Biotech, Co., Ltd, Hangzhou, China) according to its protocols.

### H&E stain and IHC

Once the tissues were harvested from mice post-treatment, the tissues were immediately fixed in formalin for 48 h in 4 °C. The collected tissues were embedded in paraffin, sectioned into about 5 μm, following standard H&E protocol. Slides were digitally scanned at Olympus IX51 Microscopy. For IHC analysis, the colons were fixed in formalin overnight and embedded in paraffin. IHC was performed to detect the TNF-α, IL-1β, and IL-6. TNF-α (ABclonal, A0277), IL-1β (ABclonal, A1112), and IL-6 (ABclonal, A0286) antibodies were used at 1:200 dilution. Visualization of stained nuclei was performed using DAB staining. Slides were digitally scanned at Olympus IX51Microscopy.

### Statistical analysis

All experiments were repeated at least three times unless otherwise indicated. Data were presented as means ± SD unless otherwise indicated. Evaluation of significance was performed using One-way ANOVA with Tukey's post hoc test. N.S. represents not significant, *p < 0.05, **p < 0.01, ***p < 0.001. Statistical analysis was performed with replications deriving from the same experiment.

## Supplementary Material

Supplementary figures and tables.

## Abbreviations

TNF: tumor necrosis factor
IBD: inflammatory bowel disease
GI: gastrointestinal
PPNP: polyphenol-PEG containing polymers self-assembled nanoparticles
TA: tannic acid
EGCG: epigallocatechin gallate
PEG: poly(ethylene glycol)
PVP: poly(N-vinyl pyrrolidone)
PDDA: poly(diallyldimethylammonium chloride)
PSS: poly(sodium 4-styrene sulfonate)
DSPE-PEG2k: 1,2-distearoyl-sn-glycero-3-phosphoethanolamine-N-[methoxy(polyethylene glycol)-2000]
CD: Crohn's disease
UC: ulcerative colitis
ROS: reactive oxygen species
PDI: polydispersity index
DLS: dynamic light scattering
TEM: transmission electron microscopy
ELISA: enzyme-linked immunosorbent assay
INF: Infliximab
