# Supplementary material for: Oral delivery of anti-TNF antibody shielded by natural polyphenol-mediated supramolecular assembly for inflammatory bowel disease therapy
Source: Theranostics. 2020 Aug 29;10(23):10808–22. doi: 10.7150/thno.47601 (PMC7482796; doi:10.7150/thno.47601)
Supplement: Supplementary file 1 — Supplementary figures and tables. [file thnov10p10808s1.pdf]

# Supplementary Materials

## Oral delivery of anti-TNF antibody shielded by natural polyphenol-mediated supramolecular assembly for inflammatory bowel disease therapy

Xinyu Wang,[1,2*] Junjie Yan,[1,2*] Lizhen Wang,[1,2] Donghui Pan,[1,2] Yuping Xu,[1,2] Fang Wang,[1] Jie Sheng,[1] Xinxin Li,[1] Min Yang[1,2]

1. NHC Key Laboratory of Nuclear Medicine, Jiangsu Key Laboratory of Molecular Nuclear Medicine, Jiangsu Institute of Nuclear Medicine. Wuxi 214063, China.

2. Department of Radiopharmaceuticals, School of Pharmacy, Nanjing Medical University, Nanjing, Jiangsu, 210029, China.

[*]X. Wang and J. Yan contributed equally to this work.

Corresponding author: Min Yang, TEL: +86-0510-85508862, FAX: +86-0510- E-mail: yangmin@jsinm.org

**Activity of α-amylase and xanthine oxidase.** Activity of α-amylase and xanthine oxidase was assayed with an activity kit (Solarbio Life Sciences, BC0615 for α-amylase and BC1095 for xanthine oxidase) using a UV–vis spectrophotometer (Perkin-Elmer, Lambda25).

**Protein encapsulation efficiency of CytC, BSA, INF in PPNP.** The fabrication process was the same with the "Preparation of protein-loaded nanoparticles" part in Experimental Section. The concentration of CytC, BSA, INF is 0.05 mg/mL. The prepared nanoparticles were filtered through a 0.05 μm filter membrane and the filtrate was collected for protein quantification. The protein concentration was quantified using BCA assay (Beyotime Biotechnology, Shanghai, China).

**Cytotoxicity assay**. HT29 cells were obtained from the Cell Bank in Shanghai Institute of Cell Biology, China. The cells were cultured at 37 °C under a humidified 5% $CO_2$ in Dulbecco's Modified Eagle's medium (DMEM) supplemented with 10% fetal bovine serum and 1% antibiotic solution (Gibco, Invitrogen, USA). The cytotoxicity of PPNP and INF@PPNP was analyzed on HT29 cells by MTT assay. The cells were seeded in 96-well plate with a density of $10^4$ cells per well and incubated overnight at 37 °C. PPNP or INF@PPNP with different concentrations were added into the wells and incubated with the cells for another 24 hours. After that, the cell viability were tested by MTT assay according to the protocol.

**In vitro TNF-α inhibition**

RAW264.7 cells were obtained from the Cell Bank in Shanghai Institute of Cell Biology, China. The cells were cultured at 37 °C under a humidified 5% $CO_2$ in Dulbecco's Modified Eagle's medium (DMEM) supplemented with 10% fetal bovine serum and 1% antibiotic solution (Gibco, Invitrogen, USA).The cells were seeded in 24-well plate with a density of $5*10^4$ cells per well and incubated overnight at 37 °C. 1 μg/mL LPS were added into the cells to activate the macrophage cells. Free INF, INF@PPNP and INF from SIG treated INF@PPNP were used to inhibit the LPS induced TNF-α production. The INF concentration is 20 μg/mL in all groups. The supernatant of cell culture were collected and the TNF-α level were tested by ELISA.

**In vitro adhesion of Cy5.5-INF@PPNP**

Plastic surfaces were coated with human recombinant transferrin or mucin from porcine stomach, respectively, to mimic inflammation and healthy mucosa. Briefly, transferrin (100 μg/ml in PBS) was added to a 24-well polystyrene plate and incubated overnight at 37°C. For mucin coating, a 3% (w/v%) solution of mucin in Hank's Balanced Salt Solution (HBSS) was added to another plate and incubated at room temperature for 2 hours with gentle shaking. 200 μl of Cy5.5-INF@PPNP was added in to wells of the transferrin-coated, mucin-coated plates and untreated plates. The plates were gently shaken at room temperature for 1 hour in the dark. Wells were then washed with PBS (3 times), and imaged using Inverted Phase Contrast Fluorescence Microscope (Olympus IX51).

**Time course analysis of INF@PPNP biodistribution.** The method for time course analysis of INF@PPNP biodistribution is similar to the "Biodistribution and inflamed colon targeting" in Experimental section. The colitis mice were administrated with Cy5.5-INF@PPNP (p.o.). The dose of INF was kept the same in all groups which are

10 mg/kg. The mice were treated with Cy5.5-INF@PPNP for 12 h and sacrificed at different time post the Cy5.5-INF@PPNP were changed by water. The tissues and organs were collected and observed under the IVIS imaging system (PerkinElmer). The images were analyzed by Living Image® software (Xenogen, CA).

**Hematological parameters and liver function analysis.** The mice were orally treated with water or PPNP (p.o.) for a week. The concentration of PPNP is 0.2 mg/mL. After treatment, the mice were sacrificed and the blood were collected for hematological parameters and liver function analysis. ALT and AST in serum were determined by ELISA.

**Colitis treatment.** Comparison of the in vivo efficacy of INF (i.v.) and INF@PPNP (p.o.) were conducted similar to "Colitis treatment" part in Experiment Section. The mice were treated by IFN (i.v.) or IFN@PPNP (p.o.) for 3 days. The concentrations of INF for IFN (i.v.) and IFN@PPNP (p.o.) are 1000 and 40 μg/mL, respectively. Colons were collected after the sacrifice of mice and the length was measured. The blood was also collected for inflammatory factors analysis by ELISA.

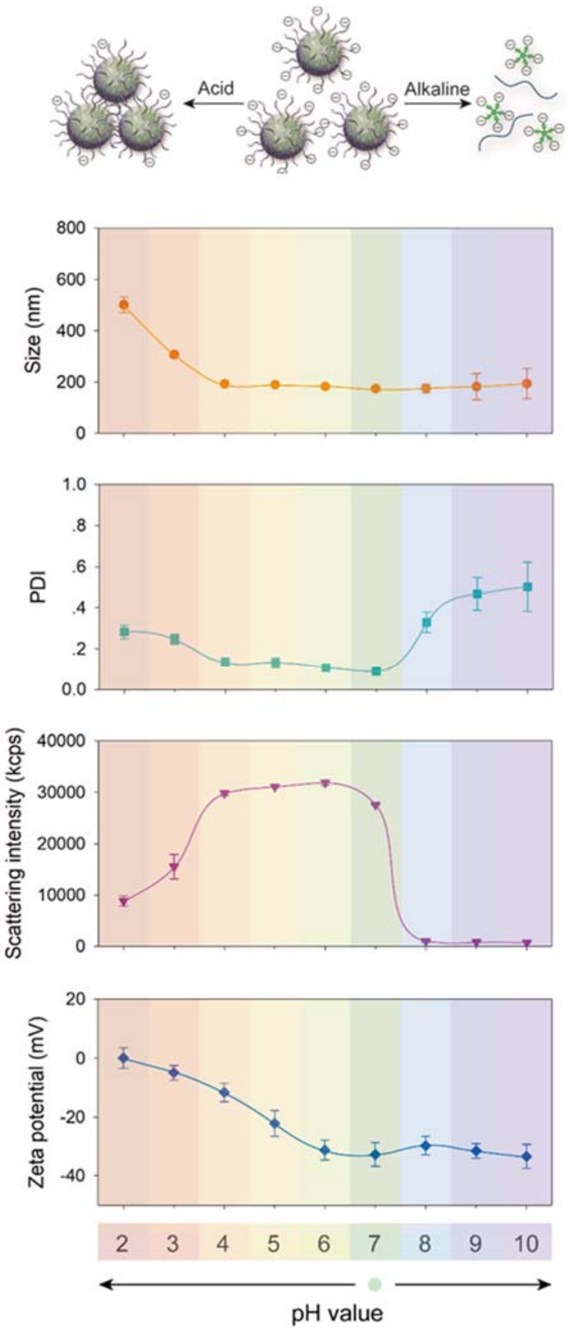


Figure S1. The size, PDI, scattering intensity, and zeta potential change of PPNP with pH titration from 7 to 2 and 7 to 10.

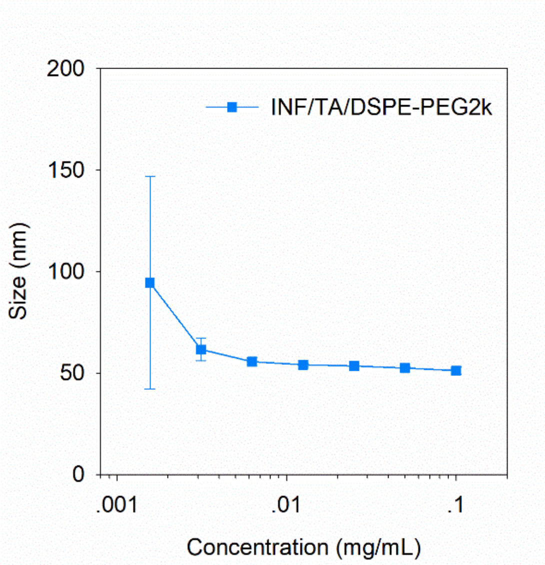


Figure S2. The average size of nanoparticles formed by INF, TA and DSPE-PEG2k at different concentration reflected by DLS. (n=3)

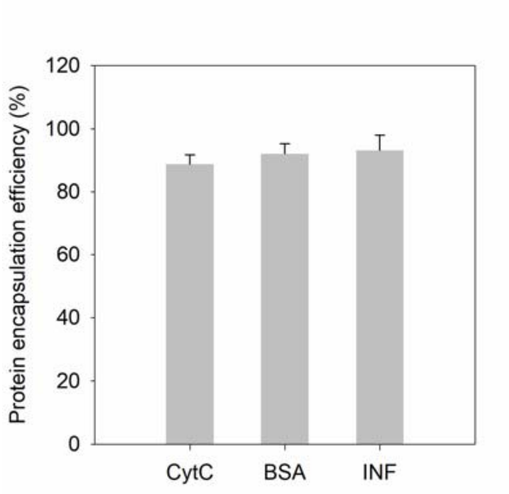


Figure S3. Protein encapsulation efficiency of CytC, BSA, INF in PPNP at a protein concentration of 0.05 mg/mL.

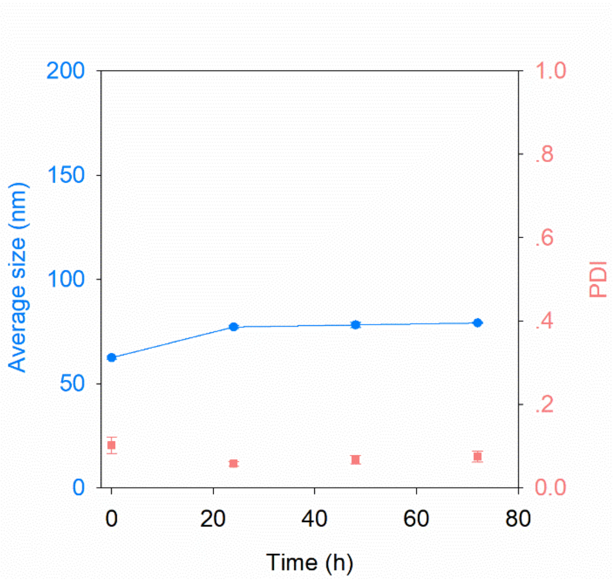

Figure S4. The average size and PDI of INF@PPNP in saline (37°C, pH 7.4) for 72 h. At 0, 24, 48 and 72 h, the size and PDI was measured by DLS.

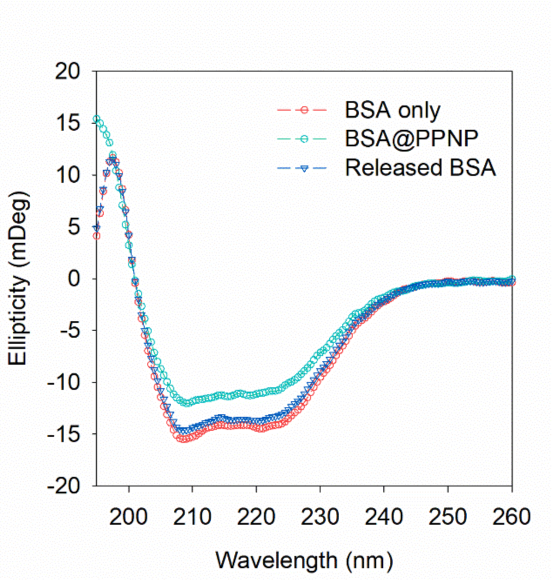

Figure S5. Circular dichroism spectra of BSA@PPNP. BSA concentration is 3 μM. The released BSA was obtained from dialysis of BSA@PPNP in a pH 9 phosphate buffer.

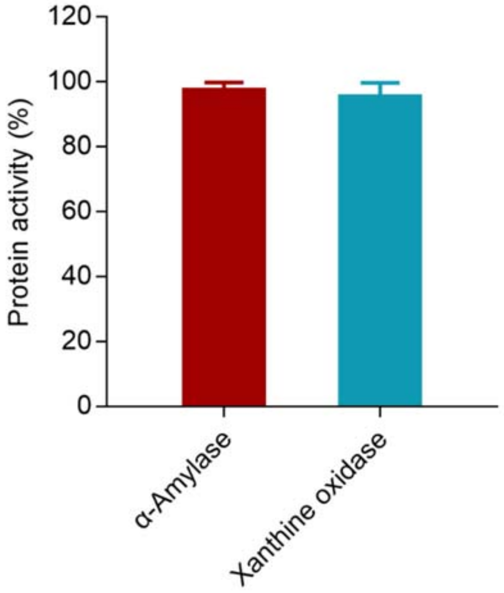


Figure S6. Protein activity of α-amylase and xanthine oxidase loaded in PPNP and recovered in basic buffer, compared to the free α-amylase and xanthine oxidase.

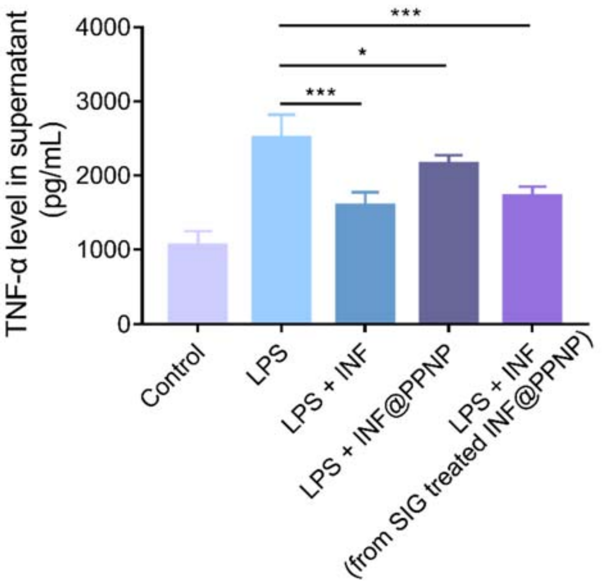


Figure S7. TNF-α level in the cultured supernatant of RAW264.7 cells determined by ELISA. The cells were activated by 1 μg/mL LPS. Free INF, INF@PPNP and INF from SIG treated INF@PPNP were used to inhibit the LPS induced TNF-α production. The INF concentration is 20 μg/mL in all groups. (n=3) *p < 0.05, ***p < 0.001 analyzed by one-way ANOVA with Tukey's post hoc test.

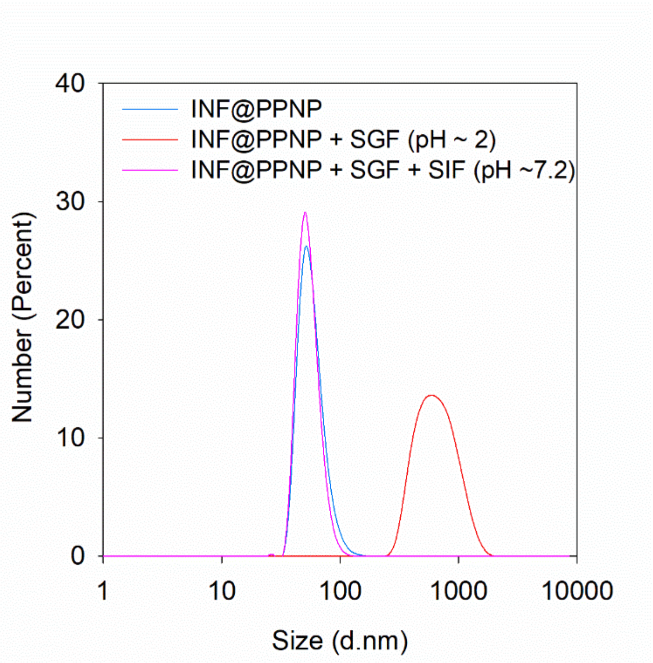


Figure S8. Size distribution of INF@PPNP incubated in SGF and SIF to simulate the pH variation process from the stomach to intestine.

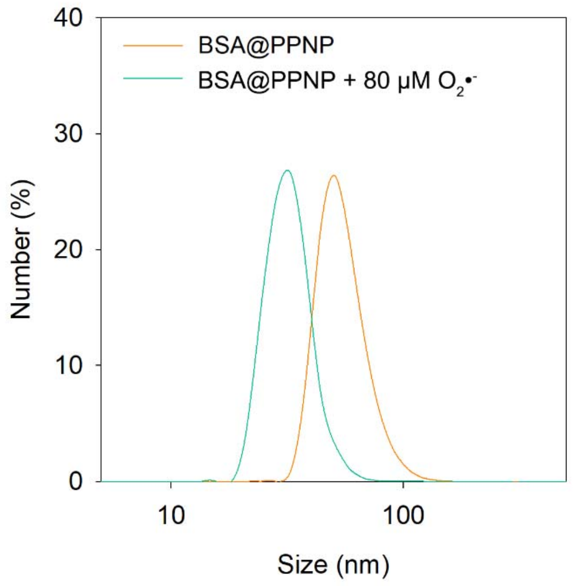


Figure S9. The size distribution of BSA@PPNP with or without 80 μM $O_2$•⁻ in SIF (pH 5.5).

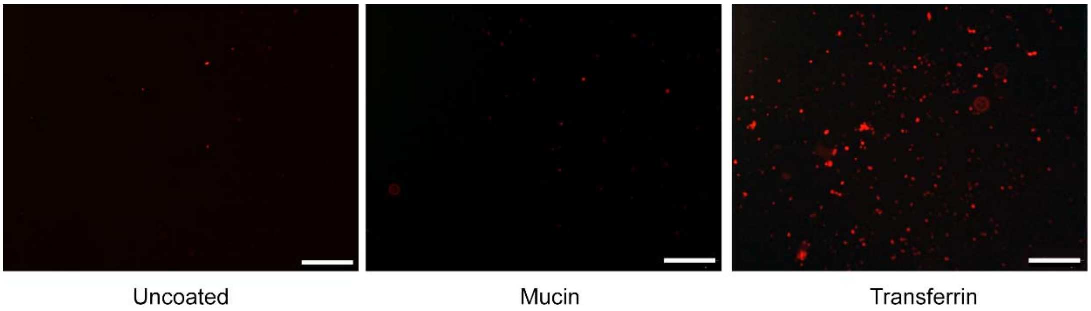


Figure S10. In vitro adhesion of Cy5.5-INF@PPNP with uncoated, mucin-coated (simulating healthy epithelium), or transferrin coated (simulating inflamed epithelium) surfaces at 37°C for 1 hour. Fluorescence images obtained after rinsing with saline. Scale bar is 100 nm.

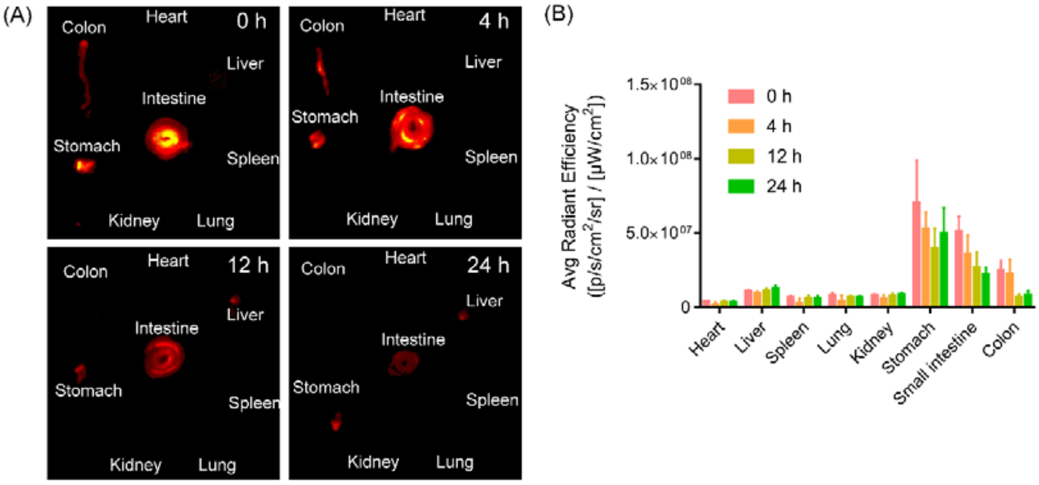


Figure S11. (A) Representative IVIS pictures of the excised organs including heart, liver, spleen, lung, kidney, stomach, small intestine and colon at various time after Cy5.5-INF@PPNP treatment. (B) Average radiant efficiency of the organs. Values are expressed as the means ± SD (n = 4).

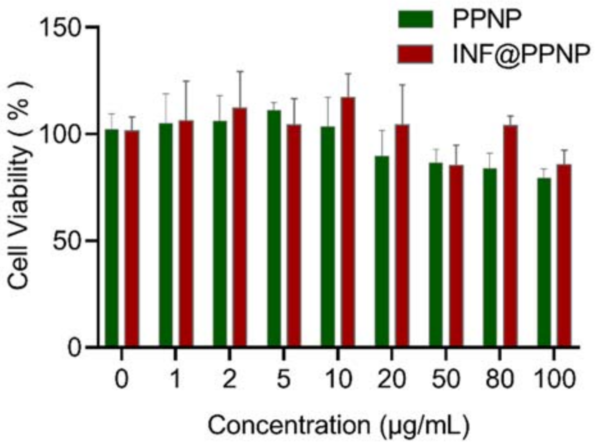


Figure S12. Cell viability of HT29 cells incubated with PPNP and INF@PPNP for 24 h. (n=4)

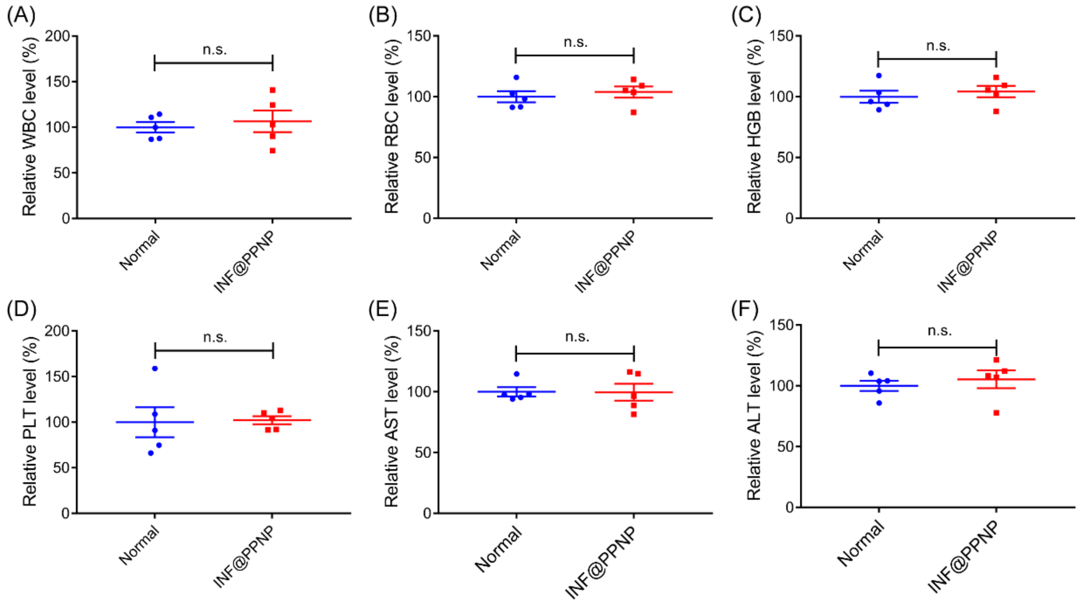


Figure S13. Relative white blood cells (WBC, A), red blood cells (RBC, B), hemoglobin (HGB, C), platelet (PLT, D) aspartate aminotransferase (AST, E) and alanine aminotransferase (ALT, F) levels in the blood of normal mice and PPNP (p.o.) treated mice. Data are presented as the mean ± SEM (n=5). n.s. $p > 0.05$ analyzed by Student's t-test.

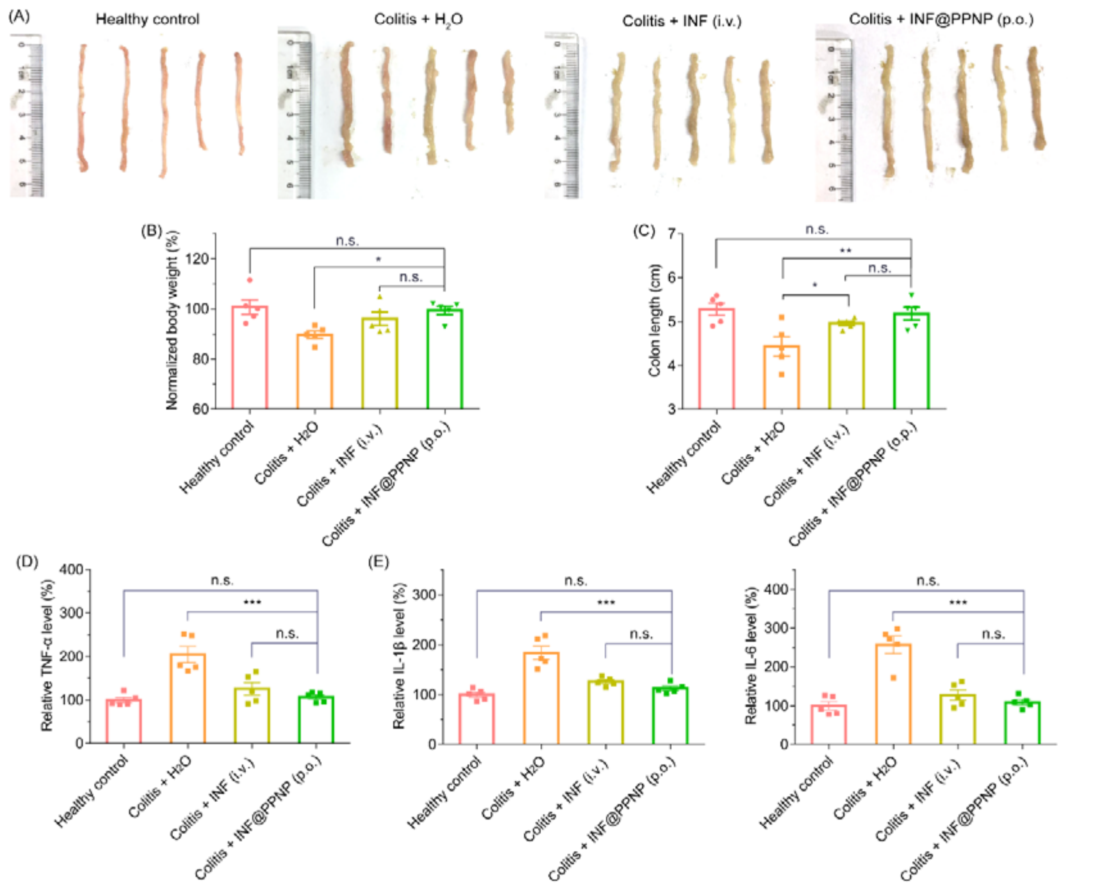


Figure S14. (A) Photos of the mice colons, (B) Body weight, and (C) colon length from various treatment groups, including healthy control, colitis, INF (i.v.) treated colitis, and INF@PPNP (p.o.) treated colitis. Relative TNF-α (D), IL-1β (E), and IL-6 (F) level in serum of the different groups. (n=5) $*p < 0.05$, $**p < 0.01$, $***p < 0.001$, n.s. $p \geq 0.05$ analyzed by one-way ANOVA with Tukey's post hoc test.
